# Duodenal‐jejunal bypass surgery activates eNOS and enhances antioxidant system by activating AMPK pathway to improve heart oxidative stress in diabetic cardiomyopathy rats

**DOI:** 10.1111/1753-0407.13516

**Published:** 2023-12-12

**Authors:** Guangwei Yang, Zitian Liu, Shuohui Dong, Xiang Zhao, Zheng Ge, Zhiqiang Cheng, Xiang Zhang, Kexin Wang

**Affiliations:** ^1^ Department of General Surgery Qilu Hospital of Shandong University Jinan China

**Keywords:** antioxidative system, diabetic cardiomyopathy, duodenal‐jejunal bypass, eNOS, oxidative stress

## Abstract

**Background:**

Diabetic cardiomyopathy is a serious complication of obesity with type 2 diabetes and is a major cause of mortality. Metabolic surgery, such as duodenal‐jejunal bypass (DJB), can effectively improve diabetic cardiomyopathy; however, the underlying mechanisms remain elusive. Oxidative stress is one of the pivotal mechanisms of diabetic cardiomyopathy. Our objective was to investigate the effect and potential mechanisms of DJB on oxidative stress in the heart of diabetic cardiomyopathy rats.

**Methods:**

High‐fat diet combined with intraperitoneal injection of streptozotocin was used to establish diabetic cardiomyopathy rats. DJB was performed on diabetic cardiomyopathy rats, and high glucose and palmitate were used to simulate diabetic cardiomyopathy in H9C2 cells in vitro. Sera from different groups of rats were used for experiments in vivo and in vitro.

**Results:**

DJB effectively improved oxidative stress and activated the adenosine monophosphate (AMP)‐activated protein kinase (AMPK) pathway to increase endothelial nitric oxide synthase (eNOS) phosphorylation level and the expression of antioxidative system‐related proteins and genes in the heart of diabetic cardiomyopathy rats. AMPK agonists and serum from DJB rats activated the AMPK pathway to increase eNOS phosphorylation level and the expression of antioxidative system‐related proteins and genes and decreased the content of reactive oxygen species in H9C2 cells, but this improvement was almost eliminated by the addition of AMPK inhibitors.

**Conclusions:**

DJB activates eNOS and enhances the antioxidant system by activating the AMPK pathway—and not solely by improving blood glucose—to improve oxidative stress in the heart of diabetic cardiomyopathy rats.

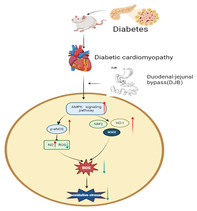

## Graphical abstract

Highlights

- This study revealed that duodenal‐jejunal bypass (DJB) attenuated oxidative stress in diabetic cardiomyopathy rats by activating the adenosine monophosphate (AMP)‐activated protein kinase (AMPK) pathway, thereby augmenting the antioxidative system and elevating the phosphorylation level of nitric oxide synthase (eNOS).
- The application of sera obtained from distinct groups and the agonist of AMPK pathway to stimulate H9C2 cells revealed that DJB intervention partially alleviated heart oxidative stress by activating the AMPK pathway, rather than solely by improving blood glucose levels.

## 1 INTRODUCTION

Over the past 3 decades, the number of people with diabetes worldwide has quadrupled, mainly due to obesity with type 2 diabetes mellitus (T2DM).^1^ Obesity with T2DM is associated with many complications, of which heart‐related complications are the main causes of death. T2DM‐related insulin resistance and hyperinsulinemia lead to metabolic disorders, such as impaired insulin signal transduction in heart tissue, leading to oxidative stress, fibrosis, hypertrophy, systolic and diastolic dysfunction, and eventually heart failure, without other recognized cardiovascular risk factors; this is known as diabetic cardiomyopathy.^2^, ^3^, ^4^, ^5^ Increasing evidence indicates that T2DM can induce a decrease in endothelial nitric oxide synthase (eNOS) phosphorylation level, thereby decreasing its activity. Inactivated eNOS decreases production of nitric oxide (NO) and increases reactive oxygen species (ROS) production, which may increase the burden of oxidative stress.^6^, ^7^ NO is indispensable for cardiovascular system homeostasis.^8^ ROS can combine with NO reducing NO bioavailability and making more serious oxidative stress, low‐NO bioavailability further predisposing toward oxidative stress, inflammation, mitochondrial dysfunction, and progressive fibrosis in T2DM‐induced cardiac injury.^7^, ^9^, ^10^ Further, the myocardial antioxidant systems, namely nuclear factor erythroid 2‐related factor 2 (NRF2)/heme oxygenase‐1 (HO‐1) and superoxide dismutase 2 (SOD2), are also damaged in diabetic cardiomyopathy, aggravating oxidative stress and causing further damage to cardiomyocytes.^9^, ^10^, ^11^ Adenosine monophosphate (AMP)‐activated protein kinase (AMPK) pathway activation can increase eNOS phosphorylation and improve the antioxidant system in heart.^12^, ^13^, ^14^ Activation of the AMPK pathway can improve oxidative stress in cardiovascular system.^9^, ^11^ Moreover, improvement of these pathways may be an effective strategy against myocardial oxidative stress in diabetic cardiomyopathy.^7^, ^10^, ^15^

In addition to traditional drug therapy, metabolic surgery is gaining acceptance as an effective treatment for T2DM and related complications, and its effectiveness in improving cardiac function and reducing cardiovascular risk in patients with T2DM has been investigated.^16^, ^17^, ^18^, ^19^, ^20^ Duodenal‐jejunal bypass (DJB) can be used to study the effect of metabolic surgery in treating T2DM in rats.^21^, ^22^ DJB can improve or even reverse pathological changes, improve cardiac function, and inhibit diabetic cardiomyopathy progression without weight loss.^23^, ^24^, ^25^ However, changes in the eNOS/NO‐related pathways, SOD2, and NRF2/HO‐1 antioxidant factors and pathways in heart tissue after DJB and their effects on oxidative stress in diabetic cardiomyopathy have not been reported.

To address this problem, we established a diabetic cardiomyopathy rat model using high‐fat diet (HFD) combined with intraperitoneal injection of streptozotocin (STZ) and performed DJB and sham operations.^26^, ^27^ Standard‐feed rats were used as a negative control to study the improvement of AMPK/eNOS/NO‐related pathways, SOD2, and NRF2/HO‐1‐related antioxidant factors and pathways in diabetic cardiomyopathy rats after DJB. Further, we established a cardiomyocyte H9C2 cell line, stimulated by high glucose (HG) and palmitate (PA) in vitro, to verify the role of related pathways and to explore whether DJB may play a role through these pathways in improving oxidative stress in the heart of diabetic cardiomyopathy rats rather than solely relying on blood glucose improvement after surgery.

## 2 MATERIALS AND METHODS

### 2.1 Animals and surgical operation

Six‐week‐old male Wistar rats were purchased from SPF Biotechnology Co., Ltd. (Beijing, China) and housed at the Animal Center of the Qilu Hospital of Shandong University. Rats were raised in a specific pathogen‐free environment with constant temperature and humidity under a 12‐h light/dark cycle and were given normal water and feed. According to a previous study by our group, 10 rats were used in each group. After 1 week of acclimatization, 10 rats were fed with normal diet continuously as the control (CON) group (*n* = 10). At the same time, other rats were fed a HFD (60% calories as fat, Xietong Biological Company, Nanjing, China) for 4 weeks to induce obesity and insulin resistance and then intraperitoneally injected with STZ (Sigma, Shanghai, China) at a dose of 35 mg/kg after 12 h of fasting to establish the type 2 diabetic rat model. One week after STZ injection, the measurement of fasting blood glucose (FBG) level (≥11.1 mmol/L) showed that the model of type 2 diabetic rats was successful. Continuous HFD feeding for another 4 weeks to increase heart damage was employed to establish the diabetic cardiomyopathy rats. Twenty diabetic cardiomyopathy rats were randomly allocated to the sham (*n* = 10) and DJB (*n* = 10) groups. A total of 30 rats were included herein. The researchers were not blinded to the group allocation during the experiment. All procedures for animals were reviewed and approved by the Animal Experimental Ethics Committee of Qilu Hospital of Shandong University, and all animal studies were conducted in accordance with the relevant ethical regulations of animal testing and research.

After fasting for 12 h, the rats in SHAM and DJB were anesthetized with 3% isoflurane, and sham and DJB operations were performed in the corresponding groups in strict accordance with the operation requirements. DJB and SHAM operations were performed according to standard procedures.^28^, ^29^, ^30^

DJB: First, a median incision was made in the upper abdomen, the duodenum was cut 1 cm away from the distal pylorus of the stomach, and the stump of the duodenum was sealed. The jejunum was then cut off at approximately 10 cm at the distal end of the ligament of Treitz, and the distal limb of the jejunum was anastomosed with the proximal duodenum. Finally, at the distal 15 cm of the duodenojejunostomy, the cholangiopancreatic limb was anastomosed end to side with the jejunum.

SHAM: The incisive and transected site was similar to that of the DJB group, and the reanastomosis was performed in situ. The operation time was the same as that in the DJB group.

After the operation, rats were fed in the cage individually, and all rats were given subcutaneous saline injection immediately, given water 24 h later, fed a liquid diet from 3 days to 1 week, and fed HFD 1 week later. Vital signs and operation‐related complications were strictly examined within 1 week. The relevant indices were recorded in accordance with the determined time. Twelve weeks after the surgery, the animals were euthanized after fasting for 12 h.

### 2.2 Physiological index

Body weight and food intake were measured before surgery and at 1, 2, 3, 4, 6, 8, 10, and 12 weeks after surgery.

### 2.3 Blood biochemistry

(FBG was measured at the tip of the tail using a blood glucose meter (Abbott, Shanghai, China) after fasting for 12 h. Before the operation and at 1, 2, and 3 months after the operation, blood was collected from the lateral canthus vein of rats under 3% isoflurane anesthesia after fasting for 12 h. Serum insulin concentration was determined using an Insulin ELISA Kit (Elabscience, Wuhan, China).

### 2.4 Oral glucose tolerance test and homeostasis model assessment of insulin resistance index

Before surgery and 3 months after the operation, oral glucose tolerance test (OGTT) was performed after fasting for 12 h. Additionally, insulin resistance homeostasis model assessment of insulin resistance (HOMA‐IR) was performed.

### 2.5 Histology and immunohistochemistry

After euthanizing the rats in each group, heart samples were taken to weigh and measure the percentage of heart mass. The paraffin sections of myocardial tissue were stained with hematoxylin and eosin, Masson's trichrome, Sirius red, wheat germ agglutinin (WGA), and col‐1 (abcam, 1:500) and col‐3 (abcam, 1:500) were detected by immunohistochemistry (Servicebio). Next, cardiomyocyte size, morphology, collagen distribution, and fibrosis were assessed. Further, fresh frozen tissue sections were stained for ROS using dihydroethidium (Sigma, Shanghai, China). Images were obtained using an Axio Scope A1 microscope (Zeiss, Oberkochen, Germany). ImageJ was used to analyze the results with the same parameters.

### 2.6 H9C2 cell culture

The H9C2 rat embryonic cardiomyocyte line was donated by the Key Laboratory of Cardiovascular Remodeling and Function of Shandong University (Jinan, China) and verified using short tandem repeat analysis. H9C2 cells were cultured in complete low‐glucose Dulbecco's modified Eagle's medium (Gibco, Shanghai, China) and were placed in a humidified incubator at 37°C and 5% CO_2_. To simulate the diabetic environment in vitro, cells were stimulated with HG + PA comprising 33.3 mM D‐glucose (Sinopharm Chemical Reagent Co., Ltd, Shanghai, China) and 0.4 mmol/L PA (Aladdin, Shanghai, China) for 48 h.^31^, ^32^ Moreover, H9C2 cells were treated with a selective AMPK agonist (20 μmol/L, A‐769662, MCE, China) for 48 h to determine the effect of activation of the AMPK pathway. Sera (10%) from the CON, SHAM, and DJB groups 12 weeks after surgery were added to H9C2 cells for 48 h to explore the effects of other improvements in the eNOS/NO pathway and antioxidant system, with the exception of changes in blood glucose levels to some extent. In addition, we used the selective AMPK inhibitor dorsomorphin (10 μmol/L, Selleck, Shanghai, China) to further verify the role of the AMPK pathway in the improvement of the eNOS/NO pathway and antioxidant system. All the simulations were performed after 12 h of starvation.

### 2.7 Protein extraction and western blot

Heart tissue proteins were extracted using a total protein extraction kit for muscles (Invent Biotechnologies, Beijing, China). Radioimmunoprecipitation assay lysate (Meilunbio, Dalian, China) was mixed with protease and phosphatase inhibitors (Solarbio, Beijing, China) to extract proteins from H9C2 cells. The concentration of extracted proteins was measured using a bicinchonic acid protein assay kit (Millipore, Billerica, MA, USA). After mixing the protein with the loading buffer (Solarbio) at 4:1, boiled protein solutions were separated by 10% sodium dodecyl‐sulfate polyacrylamide gel electrophoresis (Meilunbio) and were transferred to a 0.22 μm polyvinylidene difluoride membrane (Millipore). After 2 h of blocking with 5% skim milk powder (Beyotime, Shanghai, China), the membranes were incubated with the following primary antibodies on a 4°C shaker overnight: anti‐ACC (Proteintech, 1:1000), anti‐p‐ACC (CST, 1:1000), anti‐AMPK (CST, 1:1000), anti‐p‐AMPK (CST, 1:1000), anti‐SIRT1 (Abcam, 1:1000), anti‐β‐actin (Invitrogen, 1:2000), anti‐eNOS (Proteintech, 1:1000), anti‐p‐eNOS (CST, 1:1000), anti‐NRF2 (Proteintech, 1:4000), anti‐HO‐1 (Proteintech, 1:2000), and anti‐SOD2 (Proteintech, 1:2000). Next, they were incubated with the secondary antibody (ZSGB‐Bio, Beijing, China) at room temperature for 2 h. After adding the mixed hypersensitive electrochemiluminescence solution (Millipore), proteins were detected using a LI‐COR Odyssey Imager (LI‐COR Biosciences, Lincoln, United States). ImageJ was for quantitative analysis of western blot results.

### 2.8 Detection of NO in myocardial tissue and H9C2 cells

Fresh myocardial tissue (20 mg) was homogenized to extract the tissue lysate, which was centrifuged at 12000 × *g* for 5 min at 4°C. The myocardial NO content was detected using a NO detection kit (Nanjing Jiancheng Biological Co., Ltd., Nanjing, China).

The NO content of H9C2 cells was detected using a NO detection kit (Nanjing Jiancheng Biological Co., Ltd., Nanjing, China).

The NO levels were normalized to the total protein concentration.

### 2.9 ROS assay of H9C2 cells

The ROS levels of H9C2 cells were detected using the Reactive Oxygen Species Assay Kit (Meilunbio, Dalian, China). Images were acquired using an Axio Scope A1 microscope (Zeiss).

### 2.10 RNA extraction and reverse transcription‐quantitative polymerase chain reaction (RT‐PCR)

An RNA extraction kit (Fastagen, Shanghai, China) was used to extract RNA from rat myocardium and H9C2 cells, and the RNA concentration was measured using a NanoDrop spectrophotometer (NanoDrop Technologies, Wilmington, United States). cDNA was synthesized using the ReverTra Ace qPCR RT kit (TOYOBO, Osaka, Japan), and RT‐PCR was performed on a Roche LightCycler480 II (Roche, Basel, Switzerland) using SYBG Green real‐time fluorescence quantitative PCR premixture (TOYOBO, Osaka, Japan). Relative mRNA quantification was performed using the ΔΔCt method, and β‐actin was used as the internal reference. The sequences of the RT‐PCR primers (Sangon, Shanghai, China) were as follows:

*β‐Actin* (forward: 5′‐CCCATCATGAGGGTTACGC‐3′.

reverse: 5′‐TTTAATGTCACGCACGATTTC‐3′),

*Sirt1* (forward: 5′‐TAGGTTAGGTGGCGAGTA‐3′.

reverse: 5′‐CAGCCTTGAAATCTGGGT‐3′),

*Nrf2* (forward: 5′‐GCCTTCCTCTGCTGCCATTAGTC‐3′.

reverse: 5′‐TGCCTTCAGTGTGCTTCTGGTTG‐3′),

*Homx1* (forward: 5′‐ACCTCCTCATTGTTATTGG‐3′.

reverse: 5′‐TACTCGCCACCTAACCTA‐3′),

*Sod2* (forward: 5′‐TCCCTGACCTGCCTTACGACTATG‐3′.

reverse: 5′‐TCGTGGTACTTCTCCTCGGTGAC‐3′).

### 2.11 Statistical analysis

The data are shown as mean ± SD and were analyzed by GraphPad Prism 9 (California, USA). Two‐way analysis of variance (ANOVA) followed by Tukey's correction between‐group analysis was used to compare variations in FBG, weight, food intake, and OGTT (A: Treatment, B: Time, AB: Intervention on rats × Time). One‐way ANOVA followed by Bonferroni correction between‐group analysis was used to compare variations in other data. *p* < .05 indicated statistical significance. Statistical details are included in the respective figure legends.

## 3 RESULTS

### 3.1 DJB improves general metabolic characteristics and cardiac hypertrophy and fibrosis in diabetic cardiomyopathy rats

**FIGURE 1:**
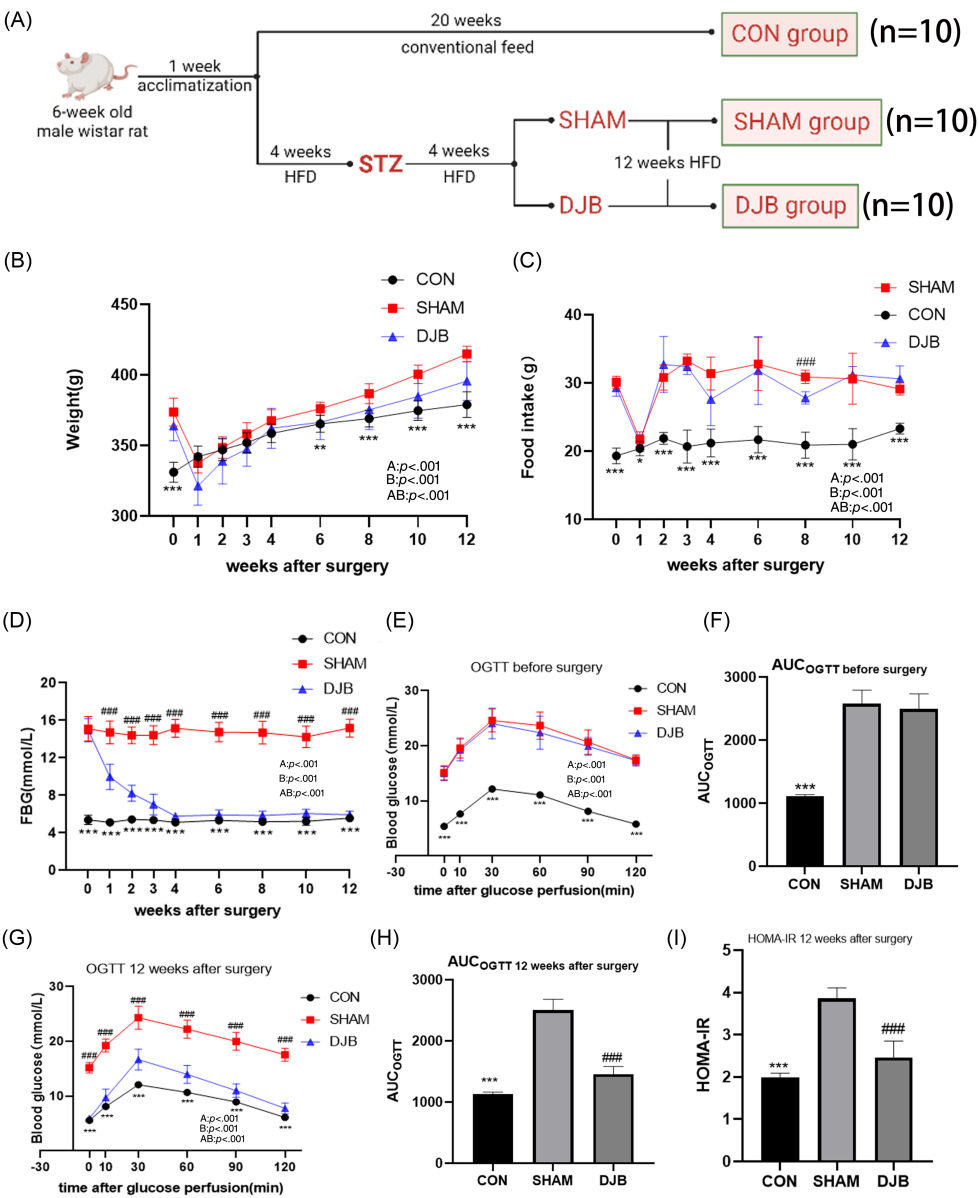
DJB improves general metabolic characteristics. (A) The flow chart of animal experiments. (B) Body weight. (C) Food intake. (D) (FBG). (E) Curves of OGTT before surgery. (F) AUC of OGTT before surgery. (G) Curves of OGTT 12 weeks after surgery. (H) AUC of OGTT 12 weeks after surgery. (I) HOMA‐IR 12 weeks after surgery. Data are presented as mean ± SD. *p < .05, **p < .01 ***p < .001 CON vs. SHAM; #*p* < .05, ##*p* < .01 ###*p* < .001 DJB vs. SHAM. *n* = 10 per group. AUC, area under the curve; CON, control group; DJB, duodenal‐jejunal bypass; FBG, fasting blood glucose; HFD, high‐fat diet; HOMA‐IR, homeostatic model assessment of insulin resistance; OGTT, oral glucose tolerance test; STZ, streptozotocin.

As shown in the curve, the body weight of rats in the DJB and SHAM groups began to decrease after surgery and began to rise from the second week (Figure 1B). There was no significant difference in the increase in body weight between the DJB and SHAM groups. Similarly, the food intake of rats in the DJB and SHAM groups decreased significantly 1 week after surgery and then gradually returned to the preoperative level (Figure 1C). The FBG levels in rats in the DJB group decreased almost to levels in rats in the CON group approximately 1 month after surgery and then remained stable, whereas there was no downward trend in FBG level in the SHAM group (Figure 1D). The OGTT showed that the glucose tolerance of the DJB group was significantly improved 12 weeks after the operation compared with that of the SHAM group, and the area under the curve decreased significantly (Figure 1E–H). Meanwhile, the HOMA‐IR values decreased after DJB compared with those in the SHAM group, suggesting that insulin sensitivity was effectively improved (Figure 1I). These results suggest that DJB can effectively improve metabolic state and glucose homeostasis.

**FIGURE 2:**
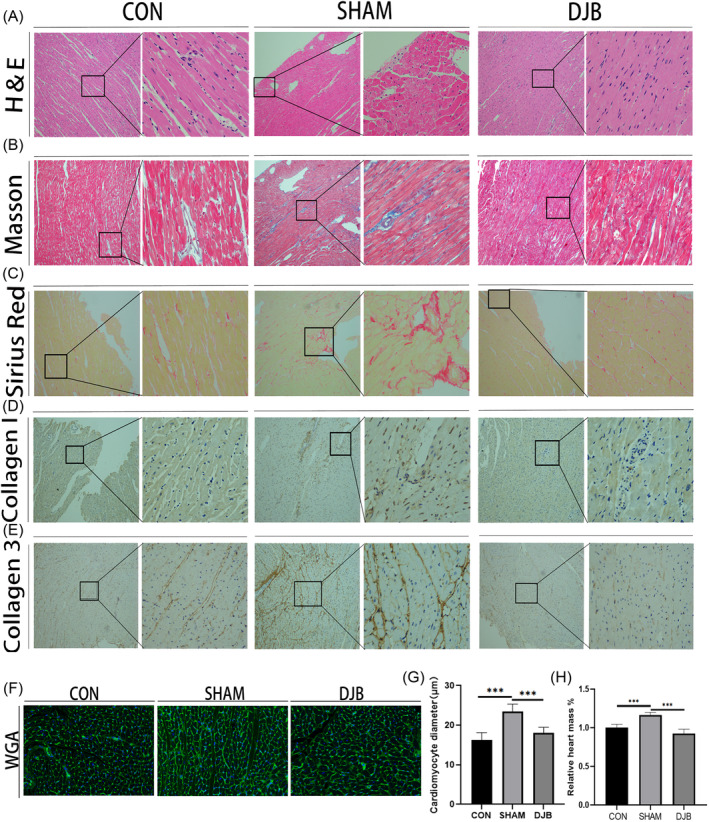
Effect of DJB on cardiac remodeling in diabetic cardiomyopathy rats. (A) H&E staining images of left ventricle (10×, 40×). (B) Masson staining of rat hearts, with dark blue staining indicating collagen fibers (10×, 40×). (C) Sirius red staining of collagen in rat hearts (10×, 40×). (D, E) Immunohistochemical staining of collagen 1 and collagen 3 (10×, 40×). (F) WGA staining images (40×). (G) Cardiomyocyte diameter. (H) Heart mass as a percentage of body weight. *p < .05. **p < .01. ***p < .001. CON, control group; DJB, duodenal‐jejunal bypass; H&E, hematoxylin and eosin; WGA, wheat germ agglutinin.

Cardiac function of diabetic cardiomyopathy rats was reportedly damaged but was improved after DJB in previous studies by our research group.^23^, ^24^, ^25^ In the present study, Hype staining and WGA staining showed that the SHAM group showed cardiomyocyte hypertrophy compared with the control group; however, hypertrophy was alleviated after DJB (Figure 2A,F,G). Heart mass as a percentage of body weight increased in diabetic cardiomyopathy rats but decreased after DJB (Figure 2H). Further, Sirius red, Masson, and immunohistochemistry staining of collagen fibers suggested a significant increase in the level of cardiac fibrosis in diabetic cardiomyopathy rats, whereas DJB effectively improved this cardiac fibrosis and reduced collagen deposition (Figure 2B–E). These results suggest that DJB can effectively improve cardiac hypertrophy and fibrosis in diabetic cardiomyopathy rats.

### 3.2 DJB ameliorates the cardiac AMPK/eNOS pathway and antioxidant system in diabetic cardiomyopathy rats and improves myocardial oxidative stress

**FIGURE 3:**
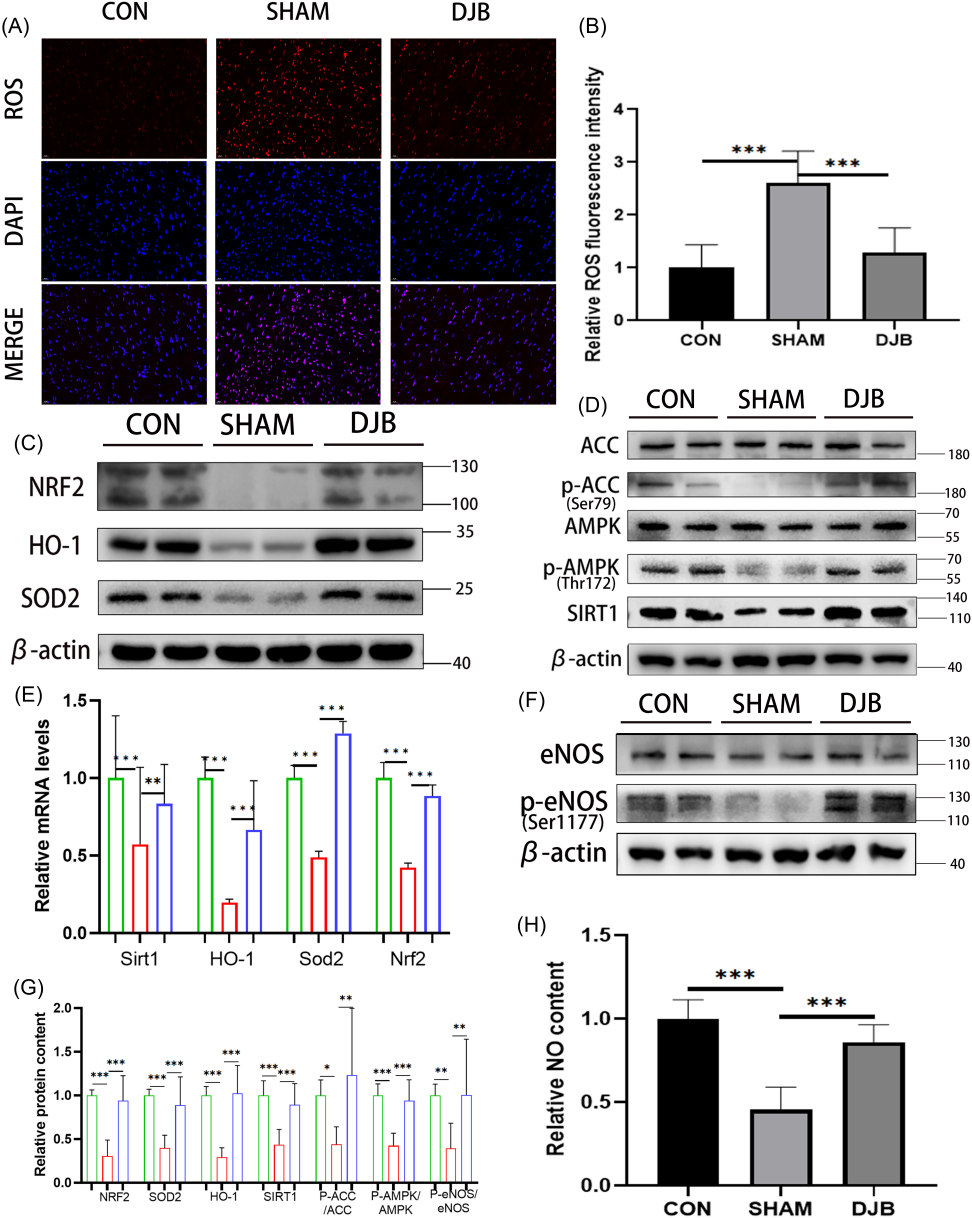
DJB ameliorates the cardiac AMPK/eNOS pathway and antioxidative system in diabetic cardiomyopathy rats and improves oxidative stress in the heart. (A) ROS fluorescence staining in the heart of three groups of rats (40×). (B) Analysis results of the relative ROS fluorescence intensity. (C) Protein levels of antioxidative system‐related factors and pathway in the heart of rats; β‐Actin was used as an internal reference. (D) Protein levels of AMPK pathway‐related molecules in the heart of rats; β‐Actin was used as an internal reference. (E) Relative mRNA levels of antioxidative system‐related factors and Sirt1 in the heart of rats; β‐Actin was used as a reference gene. (F) Phosphorylation levels of eNOS, (G) relative protein content, and (H) NO content in the heart muscle of rats 12 weeks after surgery. Data are presented as mean ± SD.*p < .05. **p < .01 ***p < .001; *n* = 10 in each group. AMPK, adenosine monophosphate‐activated protein kinase; CON, control group; DJB, duodenal‐jejunal bypass; eNOS, endothelial nitric oxide synthase; HO‐1, heme oxygenase‐1; NO, nitric oxide; NRF2, nuclear factor erythroid 2‐related factor 2; ROS, reactive oxygen species; SOD2, superoxide dismutase 2.

To detect oxidative stress pressure, we performed ROS fluorescence staining in the myocardium of the three groups of rats (Figure 3A,B). The oxidative stress pressure of diabetic cardiomyopathy rats was higher than that of normal rats, and this condition was effectively improved after DJB. Moreover, western blot results showed that the expression of AMPK pathway‐related molecules was significantly improved after DJB (Figure 3D), and the eNOS phosphorylation level (Figure 3F,G) and NO content were significantly increased (Figure 3H). Additionally, the SOD2 and NRF2/HO‐1‐related pathways, which play an important role in ROS removal, were significantly improved after DJB, which promoted the scavenging ability for ROS and improved cardiac oxidative stress (Figure 3C). Real‐time RT‐PCR showed that the relative transcriptional levels of *Sirt1*, *Nrf2*, *Homx‐1*, and *Sod2* were consistent with their corresponding protein levels (Figure 3E). These results showed that DJB can improve oxidative stress in the heart of diabetic cardiomyopathy rats by ameliorating the AMPK/eNOS pathway and antioxidant system.

### 3.3 Activation of the AMPK pathway can improve eNOS activity and antioxidant system of H9C2 cell line in the HG + PA environment

**FIGURE 4:**
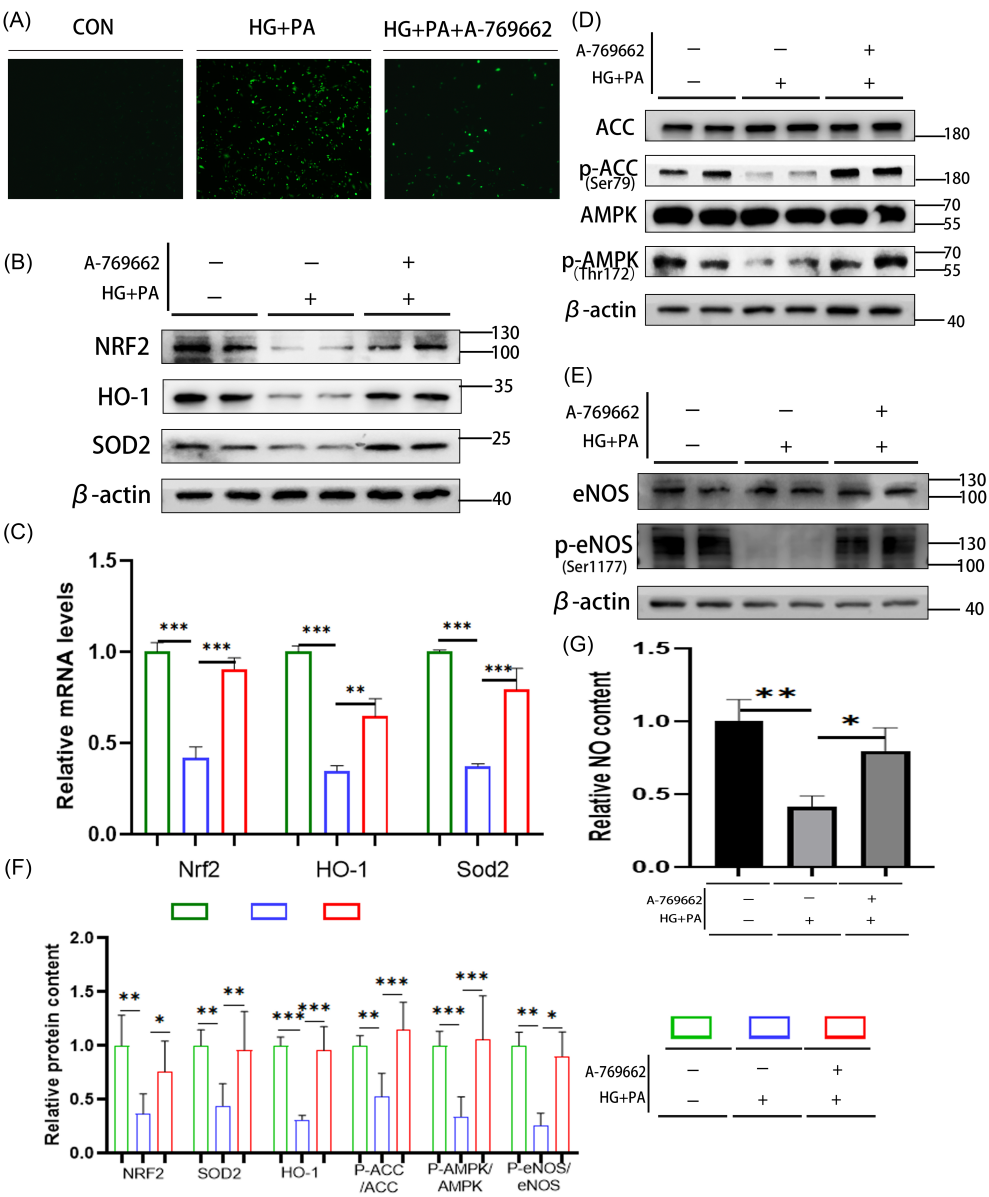
Activation of the AMPK pathway can improve eNOS activity and antioxidant system in H9C2 cells in the HG + PA environment. (A) ROS staining in H9C2 cells, shown by green fluorescence (10×). (B) Protein levels of antioxidative system‐related factors were analyzed in H9C2 cells; β‐Actin was used as an internal reference. (C) Relative mRNA levels of antioxidation system‐related factors and Sirt1 in rats; β‐Actin served as a reference gene. (D) Protein levels of AMPK pathway‐related molecules in H9C2 cells; β‐Actin was used as an internal reference. (E) Phosphorylation levels of eNOS in H9C2 cells; β‐Actin was used as an internal reference. (F) Relative protein content and (G) NO content in H9C2 cells. Data are presented as mean ± SD. *p < .05. **p < .01. ***p < .001 *n* = 3 in each group. AMPK, adenosine monophosphate‐activated protein kinase; CON, control group; DJB, duodenal‐jejunal bypass; eNOS, endothelial nitric oxide synthase; HG, high glucose; HO‐1, heme oxygenase‐1; NO, nitric oxide; NRF2, nuclear factor erythroid 2‐related factor 2; PA, palmitate; ROS, reactive oxygen species; SOD2, superoxide dismutase 2.

To verify the function of the AMPK pathway, HG + PA was used to stimulate H9C2 cells to simulate the HG and high‐fat environment of diabetic cardiomyopathy. A‐769662 was used to stimulate H9C2 cells for 48 h to activate the AMPK pathway. ROS staining showed that ROS activity in H9C2 cells increased significantly in the HG + PA group but decreased significantly after AMPK pathway activation (Figure 4A). Western blotting showed that in the HG + PA environment, the activity of the AMPK pathway decreased (Figure 4D) and eNOS phosphorylation decreased, decreasing the activity of eNOS (Figure 4E). The NO detection results showed that the NO content decreased (Figure 4F). Further, the antioxidant system, such as the SOD2 and NRF2/HO‐1 pathways, also decreased (Figure 4B). Real‐time RT‐PCR showed that the relative transcriptional levels of *Nrf2*, *Homx‐1*, and *Sod2* were consistent with their corresponding protein levels (Figure 4C). After activation of the AMPK pathway, western blotting showed that the protein levels of AMPK/eNOS were significantly increased (Figure 4D,E), and the NO content was significantly increased (Figure 4F). The antioxidant system‐related protein SOD2 and NRF2/HO‐1 pathways were also significantly increased (Figure 4B). Real‐time RT‐PCR showed that the relative transcriptional levels of *Nrf2*, *Homx‐1*, and *Sod2* were consistent with their corresponding protein levels (Figure 4C). The eNOS phosphorylation level and the antioxidant system were damaged in the HG + PA environment, while activation of the AMPK pathway reversed this. The above results verified the function of the AMPK pathway and related molecules in vitro.

### 3.4 Effects of CON, SHAM, and DJB group rat sera on AMPK/eNOS pathway and antioxidant system of H9C2 cells in the HG + PA environment

**FIGURE 5:**
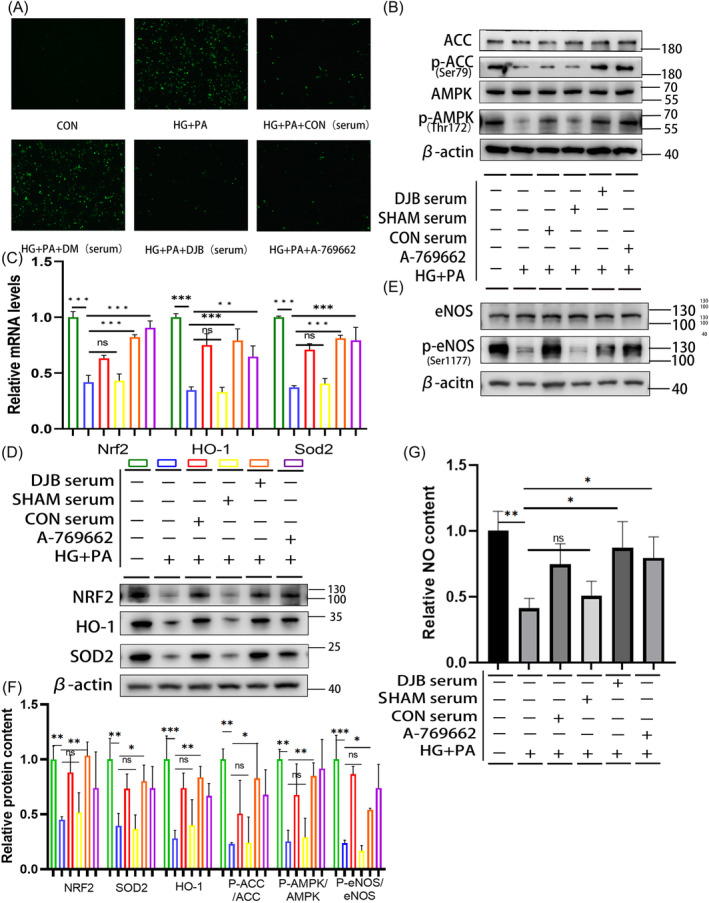
Effects of CON, SHAM, and DJB group sera on the AMPK/eNOS pathway and antioxidant system in H9C2 cells in the HG + PA environment. (A) ROS staining in H9C2 cells, shown by green fluorescence (10×). (B) Protein levels of AMPK pathway‐related molecules in H9C2 cells; β‐Actin was used as an internal reference. (C) Relative mRNA levels of antioxidation system‐related and Sirt1 genes in rats; β‐Actin served as a reference gene. (D) Protein levels of antioxidative system‐related factors in H9C2 cells; β‐Actin was used as an internal reference. (E) Phosphorylation levels of eNOS in H9C2 cells; β‐Actin was used as an internal reference. (F) Relative protein content and (G) NO content in H9C2 cells. Data are presented as mean ± SD. *p < .05. **p < .01. ***p < .001; *n* = 3 in each group. AMPK, adenosine monophosphate‐activated protein kinase; CON, control group; DJB, duodenal‐jejunal bypass; DM, diabetes mellitus; eNOS, endothelial nitric oxide synthase; HG, high glucose; HO‐1, heme oxygenase‐1; NO, nitric oxide; NRF2, nuclear factor erythroid 2‐related factor 2; PA, palmitate; ROS, reactive oxygen species; SOD2, superoxide dismutase 2.

To further verify the connection between DJB and the AMPK pathway, eNOS phosphorylation and the antioxidant system were assessed. Rat sera from the CON, SHAM, and DJB groups were used to stimulate H9C2 cells in the HG + PA environment. The external HG + PA environment almost eliminated the changes in blood glucose levels in the serum of rats in the different groups. The results showed that under stimulation with the SHAM group serum, the oxidative stress of H9C2 cells in the HG + PA environment did not improve and was even aggravated (Figure 5A). Meanwhile, western blotting showed that the levels of AMPK pathway‐related proteins also did not improve (Figure 5B). The eNOS phosphorylation level and those of antioxidant system‐related pathway proteins were similar to those in the HG + PA group, with no significant improvement (Figure 5E). Further, the NO content decreased (Figure 5F). Real‐time RT‐PCR showed that the relative transcriptional levels of *Nrf2*, *Homx‐1*, and *Sod2* were consistent with their corresponding protein levels (Figure 5C). However, under stimulation with the DJB and CON group sera, oxidative stress resulting from the HG + PA environment significantly improved. ROS staining showed that the level of ROS was decreased (Figure 5A). Western blotting showed that the AMPK pathway was activated (Figure 5B), and the eNOS phosphorylation level increased (Figure 5E). The NO content also increased (Figure 5F). Simultaneously, antioxidant system‐related proteins were significantly improved (Figure 5D). Real‐time RT‐PCR showed that the transcriptional levels of *Nrf2*, *Homx‐1*, and *Sod2* were consistent with the corresponding protein levels (Figure 5C).

### 3.5 Improvement of oxidative stress in H9C2 cells in HG + PA environment by serum of DJB rats depends on activation of the AMPK pathway

**FIGURE 6:**
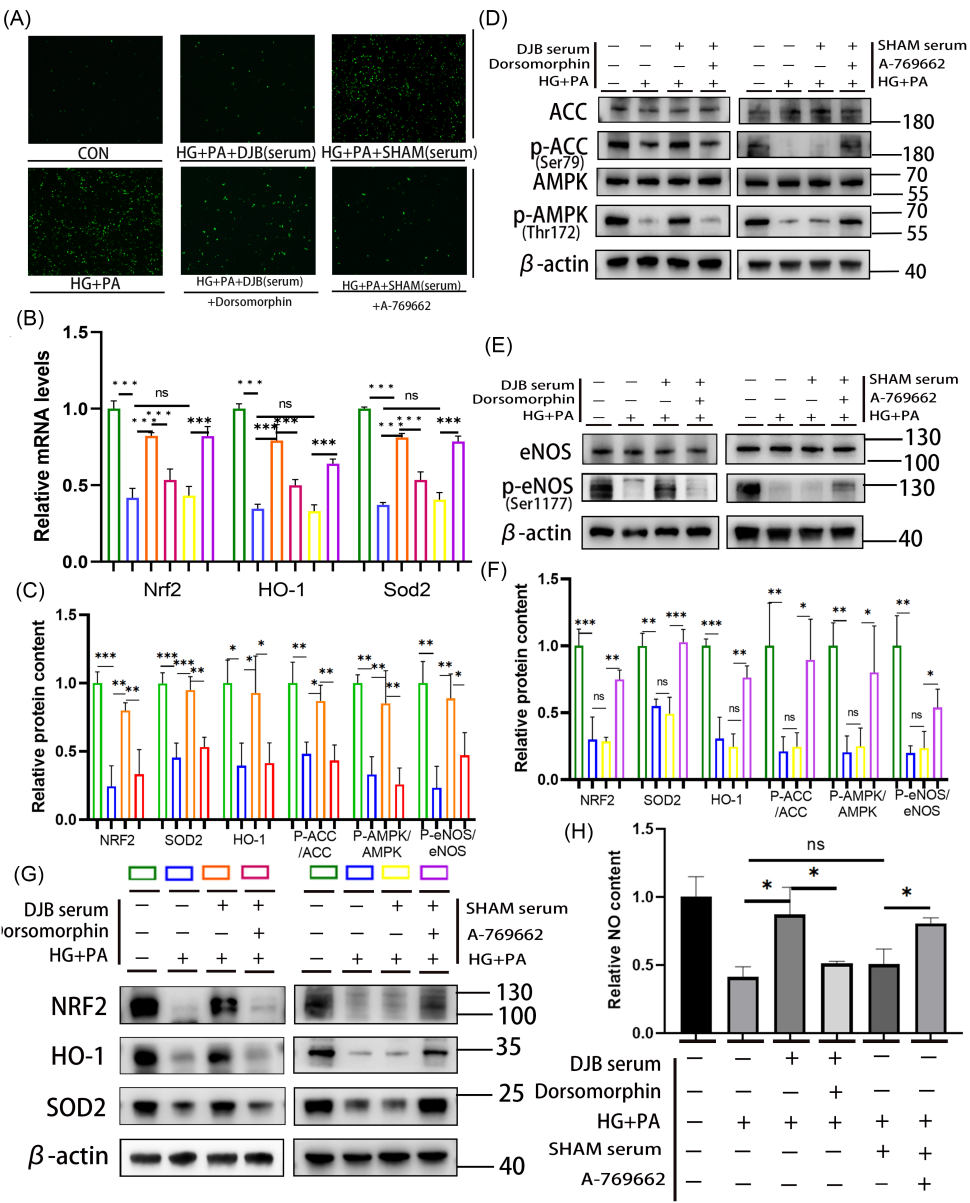
Improvement of oxidative stress in H9C2 cells in the HG + PA environment by the serum of DJB rats depends on the activation of the AMPK pathway to a certain extent. (A) ROS staining in H9C2 cells, shown by green fluorescence (10×). (B) Relative mRNA levels of antioxidation system‐related genes in rats. β‐Actin served as a reference gene. (C) Relative protein content and (D) protein levels of AMPK pathway‐related molecules in H9C2 cells; β‐Actin was used as an internal reference. (E) Phosphorylation levels of eNOS in H9C2 cells; β‐Actin was used as an internal reference. (F) Relative protein content and (G) protein levels of antioxidative system‐related factors in H9C2 cells; β‐Actin was used as an internal reference control. (H) NO content in H9C2 cells. Data are presented as mean ± SD. *p < .05. **p < .01 ***p < .001; *n* = 3 in each group. AMPK, adenosine monophosphate‐activated protein kinase; CON, control group; DJB, duodenal‐jejunal bypass; eNOS, endothelial nitric oxide synthase; HG, high glucose; HO‐1, heme oxygenase‐1; NO, nitric oxide; NRF2, nuclear factor erythroid 2‐related factor 2; PA, palmitate; ROS, reactive oxygen species; SOD2, superoxide dismutase 2.

To verify the effect of the AMPK pathway, the AMPK inhibitor dorsomorphin combined with DJB group serum and AMPK agonist combined with SHAM group serum were used to stimulate the AMPK pathway in H9C2 cells in the HG + PA environment. The results showed that the reduction in ROS as well as the improvement in the AMPK/eNOS pathway, NO content, and antioxidant system was inhibited after adding the AMPK inhibitor (Figure 6A,C–F). By contrast, the decrease in ROS and the improvement of the AMPK/eNOS pathway and antioxidative system were further improved after adding the AMPK agonist combined with the SHAM group serum in H9C2 cells in the HG + PA environment (Figure 6A,C–F). Real‐time RT‐PCR showed that the relative transcriptional levels of *Nrf2*, *Homx‐1*, and *Sod2* were consistent with their corresponding protein levels (Figure 6B).

## 4 DISCUSSION

Diabetic cardiomyopathy, one of the most serious complications of T2DM, has always been one of the leading causes of death in patients with T2DM.^4^, ^5^ In addition to traditional drug therapy, metabolic surgery has increasingly become an important treatment for T2DM and its complications.^16^, ^19^ Metabolic surgery can effectively reduce the risk of cardiovascular diseases, such as hypertension, coronary heart disease, and heart failure, in patients with T2DM and effectively reduce the mortality of patients.^33^, ^34^, ^35^

DJB can effectively improve T2DM and its complications in patients and rat models.^28^, ^36^, ^37^ Our research group has shown that DJB can improve the myocardial function of diabetic cardiomyopathy rats possibly via improving cardiac endoplasmic reticulum stress, promoting Ca2+ homeostasis, decreasing myocardial autophagy, and restoring myocardial glucose uptake through myocardial glucose translocation.^23^, ^24^, ^25^ Moreover, oxidative stress is considered one of the important phenomena involved in diabetic cardiomyopathy pathogenesis, and the production and elimination of ROS are important indicators of the degree of oxidative stress in cardiomyocytes. Here, we found that DJB effectively improved the metabolism of T2DM rats, consistent with previous studies. Activation of eNOS/NO can improve oxidative stress in cardiovascular system.^38^, ^39^ In this study, ROS staining suggested that significant improvement in myocardial oxidative stress took place after DJB. We also found that eNOS phosphorylation increased after DJB showing that activity of eNOS and production of NO were improved, which may play a role in reducing ROS. NO also play an important role in maintaining cardiovascular system homeostasis besides oxidative stress.^7^, ^8^ Increasing NO can also alleviate T2DM‐induced heart injury by improving inflammation, mitochondrial dysfunction, hypertrophy, and progressive fibrosis. As an important antioxidant, SOD2 and NRF2/HO‐1 play indispensable role in reducing ROS in cardiovascular system.^9^, ^11^ In the present study, expression of SOD2 and NRF2/HO‐1 pathway‐related molecules all increased after DJB in heart of T2DM rats, suggesting that DJB may improve the antioxidative system in the heart of T2DM rats. One study showed that sleeve gastrectomy (SG) can increase expression of SOD2 and NRF2/HO‐1 pathway in the heart of T2DM rats, which showed the improvement of antioxidative system.^40^ These results suggest that although SG and DJB are different operations, they may share similar pathways associated with the cardiac antioxidative system.

Activation of AMPK pathway is very important for improving metabolism and can improve oxidative stress in heart, increasingly becoming an important therapeutic target.^9^, ^41^, ^42^ Some drugs, metformin for example, can activate the AMPK pathway to get cardiovascular benefits and alleviate T2DM‐induced injury in heart.^43^, ^44^, ^45^ In this study, we also found that the myocardial AMPK pathway was significantly activated after DJB. Similarly, AMPK pathway activation can promote eNOS phosphorylation and improve the antioxidant system,^46^, ^47^, ^48^, ^49^ suggesting that the improvement of oxidative stress by DJB may depend on the AMPK pathway in the present study. In the context of a HG + PA environment, our study demonstrates the potential feasibility of enhancing eNOS phosphorylation and the antioxidative system (SOD2, NRF2/HO‐1) through the activation of the AMPK pathway in H9C2 cells. These results reflected the effect of the AMPK pathway on eNOS activation and antioxidant system in cardiomyocytes to some extent.

The main cause of diabetic cardiomyopathy is diabetic hyperglycemia; however, blood glucose control alone may be limited in alleviating cardiovascular diseases.^50^, ^51^ Therefore, the potential mechanism of DJB in improving cardiac oxidative stress in diabetic cardiomyopathy rats may be related to the changes in oxidative stress‐related pathways mentioned earlier, rather than just relying on the improvement of blood glucose and other metabolic indices after DJB. Previous studies on metabolic surgery in diabetic cardiomyopathy rats found some improvements in metabolic pathways only after DJB or SG; however, they were unable to determine whether the effect of DJB or SG on the heart of diabetic cardiomyopathy rats was related to mechanisms induced by the surgery itself or was secondary to the improvement of blood glucose after surgery.^23^, ^40^, ^52^ To explore this, we added the sera of different groups of rats to H9C2 cells in the HG + PA environment. The contents of glucose and lipids in H9C2 cells were much higher than those in the serum of rats, indicating that there was no significant change in the concentration of glucose and lipids in H9C2 cells after the addition of sera from the different groups. We also aimed to simulate the myocardial environment of SHAM and DJB rats and, to some extent, eliminate the effects of changes in blood glucose levels. Stimulating H9C2 cells with the DJB group serum or CON serum in the HG + PA environment showed that this serum exhibited a general trend of promoting eNOS phosphorylation, SOD2 expression, and the NRF2/HO‐1 pathway; decreasing ROS; and improving oxidative stress in these cells, whereas the SHAM group serum showed the opposite trend. To explore the role of the AMPK pathway, we added AMPK inhibitors to H9C2 cells supplemented with the DJB group serum in the HG + PA environment. The results suggested that the improvement is dependent, at least partially, on the activation of the AMPK pathway. The results obtained with the SHAM group serum combined with AMPK agonist in the HG + PA environment also showed the significance of the AMPK pathway in the improvement of oxidative stress and the overall trend of AMPK pathway alterations in the SHAM group. These results may show that the DJB serum make the tendency trend to the CON serum rather than SHAM serum, which can activate the AMPK related pathway and improve oxidative stress in myocardial cells with some potential mechanisms.

We confirmed that DJB effectively improved oxidative stress in heart of T2DM rats and provided evidence that the improvement of heart oxidative stress in diabetic cardiomyopathy rats by DJB may be due to improvement of the expression of these molecules, which depends on AMPK pathway activation. This improvement also appears to be partially independent of the DJB‐induced improvement of blood glucose in diabetic cardiomyopathy rats, providing possible solutions to the problems in previous metabolic surgery studies. However, our present results only showed that there may be one or a group of factors in serum that can play a role in improvement of oxidative stress in heart of T2DM rats depending on AMPK pathway after DJB, we cannot confirm the specific factor or factors due to the complex composition of serum. Some factors such as glucagon‐like peptide‐1, glucose‐dependent insulinotropic polypeptide, or fibroblast growth factor, which play important roles in improvement of T2DM metabolism after metabolic surgery such as DJB and SG,^53^, ^54^, ^55^, ^56^, ^57^, ^58^ have been reported to have the ability to activate AMPK pathway^59^, ^60^, ^61^, ^62^, ^63^ and may act as candidate factors being responsible for the improvement of oxidative stress. Further research is still warranted.

In summary, we found that DJB can improve oxidative stress in the heart of diabetic cardiomyopathy rats with the potential mechanism involving activation of the AMPK pathway and further improvement of eNOS/NO pathway, SOD2 expression, and the NRF2/HO‐1 pathway, not solely depending on the improvement of blood glucose.

## AUTHOR CONTRIBUTIONS

Conceptualization, Kexin Wang and Xiang Zhang; methodology, Guangwei Yang; software, Guangwei Yang; validation, Guangwei Yang, Zitian Liu and Xiang Zhao; formal analysis, Guangwei Yang; investigation, Guangwei Yang; resources, Shuohui Dong and Zheng Ge; data curation, Zitian Liu; writing—original draft preparation, Guangwei Yang; writing—review and editing, Guangwei Yang; visualization, Shuohui Dong and Zhiqiang Cheng; supervision, Xiang Zhan and Kexin Wang; project administration, Xiang Zhang and Kexin Wang; funding acquisition, Kexin Wang. All authors have read and agreed to the published version of the manuscript.

## DISCLOSURE

The authors declare no conflict of interest.

## INSTITUTIONAL REVIEW BOARD STATEMENT

The animal study was approved by the Scientific Research Ethics Committee of Qilu Hospital of Shandong University. (approval document no. DWLL‐2021‐033, Jinan, China).

## Data Availability

The data presented in this study are available on request from the corresponding author.
